# Quality and quantity of leaf litter: Both are important for feeding preferences and growth of an aquatic shredder

**DOI:** 10.1371/journal.pone.0208272

**Published:** 2018-12-12

**Authors:** Rebeca Arias-Real, Margarita Menéndez, Meritxell Abril, Francesc Oliva, Isabel Muñoz

**Affiliations:** 1 Department of Evolutionary Biology, Ecology and Environmental Sciences, Faculty of Biology, University of Barcelona, Barcelona, Spain; 2 BETA Technological Centre, University of Vic-Central University of Catalonia, Vic, Spain; 3 Department of Genetics, Microbiology and Statistics, Faculty of Biology, University of Barcelona, Barcelona, Spain; Universidade Regional Integrada do Alto Uruguai e das Missoes, BRAZIL

## Abstract

The study of leaf litter as a resource for shredders has emerged as a key topic in trophic links in ecology. However, thus far, most studies have emphasized the leaf quality as one of the main determinants of shredder behaviour and growth without simultaneously considering the leaf quantity availability. Nevertheless, the combined effects of leaf quantity and quality on shredder behaviour and growth is particularly crucial to further understand how ecosystem functioning may respond to the increasing flow intermittency due to climate change. In this study, we explore how changes in the leaf litter quality and quantity influence the feeding preferences and growth of an invertebrate shredder (*Potamophylax latipennis*). To do so, we used black poplar leaves conditioned in two streams with different flow regimens as a food resource. Afterwards, using a microcosm approach, we offered leaf discs that varied in terms of leaf quantity and quality to *P*. *latipennis*. Our results showed that flow intermittency had a negative effect on the quality of the food resource, and a lower quality had a negative effect on the consumption and growth rates of *P*. *latipennis*. Furthermore, we found that *P*. *latipennis* fed selectively on higher quality leaves even though the availability (quantity) of this resource was lower. In the context of climate change, with higher aridity/drier conditions/scenarios, our findings suggest that a decrease in the availability (quantity) of high-quality resources could potentially threaten links in global fluvial food webs and thus threaten ecosystem functioning.

## Introduction

Rivers that naturally and periodically cease to flow in time and/or space are termed intermittent rivers (IRs) and are recognized as the most common fluvial ecosystem around the world [1]. The seasonal flow variability in IRs is the most important factor that determines their functioning; for instance, this variability determines the nutrient dynamics or hydrological connectivity essential for community dispersion [2].

As a result of the increasing aridification caused by climate change, many streams are expected to become IRs, experiencing greater variability in their water flow, which may eventually lead to complete flow disruption [3]. Despite the importance and increasing abundance of IRs, the effects of changes in water flow on biodiversity and ecosystem functioning are still largely unknown [4].

Organic matter decomposition is a key ecosystem process that influences the cycling of nutrients and energy flow to higher trophic levels [5,6]. Microbial decomposers (fungal and bacterial communities) and invertebrate shredders are the biological drivers of organic matter decomposition. Among the microbial decomposers, aquatic hyphomycetes (fungal community) are considered the first colonizers and main drivers of microbial decomposition in its first stages [7], which constitutes an important trophic link between leaf litter and shredders [8]. Aquatic hyphomycetes improve the palatability and nutritional quality (increasing the proteins, lipids and carbohydrates due to the characteristics of the fungi themselves) of leaf litter by transforming recalcitrant polymers into more labile molecules via their enzyme capabilities [9,10] and increasing the nitrogen and phosphorus concentrations of leaf litter via the accumulation of fungal mycelia [11,12]. This leaf transformation is crucial for shredders, which need a critical amount and balance of inorganic and organic elements for growth, reproduction and maintenance [9]. Moreover, the shredders cannot synthesize some essential components (e.g. essential fatty acids) and must therefore acquire them from their diet [13]. Consequently, shredders tend to consume the most optimal resource, which is the most energetic and nutrient-rich food available.

Generally, flow reduction affects the communities of aquatic hyphomycetes, which are particularly vulnerable to desiccation stress, especially those that are not adapted to flow reduction [14]. Some studies have shown that during flow reduction, the communities of aquatic hyphomycetes can experience a shift in fungal richness and composition and alterations in their enzymatic activity, such as summer drying conditions inhibiting lignocellulolytic enzyme activities [10,15,16]. These changes in the communities of aquatic hyphomycetes coupled to changes in the abiotic conditions of streams under flow reduction (e.g., decreasing the dissolved oxygen content and increasing the water temperature and conductivity) are expected to affect organic matter decomposition and the feeding links to higher trophic levels [17,18].

In addition, flow reduction also affects the riparian vegetation, causing early leaf abscission [19]. This may lead to temporal and spatial changes in this basal resource for aquatic hyphomycetes and shredders. Despite its potential implications for the organic matter cycle, the effects of decreasing the availability of high-quality resources, such as leaf litter, on consumers is still poorly studied.

To date, studies have focused on leaf quality to explain invertebrate shredder behaviour and growth [20–22]. For example, Frost et al. [23,24] have shown that shredders compensated for nutrient limitations by increasing feeding rates or selectively feeding on resources with more nutritious properties. Nevertheless, little is known regarding the effect of combined changes in leaf quantity and quality on the feeding preferences of invertebrate shredders and their growth. This knowledge is particularly crucial for understanding how biodiversity (e.g., aquatic hyphomycetes community) and ecosystem functioning (e.g., organic matter decomposition and its implications on stream food webs [22]) may respond to ongoing effects of climate change.

In line with this information, we address this knowledge gap herein by exploring how changes in both the leaf litter quality and quantity affect or determine the feeding preferences and growth of an invertebrate shredder using field and microcosm approaches. To do so, we first assessed the influence of flow intermittency on the leaf litter quality (thought fungal biomass, C:N ratios and total lipid content) and the composition of the associated community of aquatic hyphomycetes. We expected that under flowing conditions (a permanent stream) the leaf litter would be of better quality (lower C:N ratios and higher fungal biomass and lipid content) than that from an IRs. Second, we explored the joint effects of leaf litter (food resource) quality and quantity on the consumption rates and growth of a shredder using microcosms. We expected that better quality food resources and availability would be correlated with higher consumption rates and growth of the shredder. Finally, we quantified the feeding preferences of the shredder, expecting that the quality rather the quantity of the resource would be more important. Therefore, even if the shredder has a larger quantity of poor-quality resources, they will actively select the best quality food.

## Materials and methods

No specific permissions were required for my locations/activities. Our study did not involve endangered or protected species.

### Leaf litter and fungal assemblages

We used black poplar leaves (*Populus nigra* L.*)* conditioned in two different streams as a food resource. We selected one permanent stream (Arbucies in the Tordera Basin, N 41° 823133 E 2° 452826; hereafter termed “A”) and one intermittent stream (Llobina in the Besos Basin, N 41° 46.011 E 2° 16.104; hereafter termed “B”) in Catalonia, Spain. Both streams are third-order streams, meaning that they have different flow regimes but similar water physicochemical characteristics [25]. Furthermore, both streams have the same geology (siliceous bedrock) and poplar (*Populus nigra* L.), alder (Alnus glutinosa (L.) Gaertner) and evergreen oak (Quercus ilex L.O) are the dominant riparian vegetation. In addition, previous studies have indicated differences in the biodiversity of aquatic hyphomycetes between these streams [26], with lower diversity in the Llobina stream (B).

To characterize the stream hydrology, in February 2016, we placed Leveloggers (Solinst Levelogger Edge, full-scale reading precision of 0.05%) on the streambed for water level and temperature recordings as a proxy to measure the presence or absence of flow. The Leveloggers were recorded at hourly intervals for one year (from February 2016 until January 2017). The recorded data were corrected with barometric pressure variations using data from Barologgers (Solinst Barologger, full-scale reading precision of 0.05%). We installed the Barologgers at each site in the riparian area to measure the atmospheric pressure changes. In addition, throughout this year, we collected samples two times (in October 2016 and January 2017) to analyse the water concentrations of nutrients (nitrate, nitrite, ammonium and soluble reactive phosphorous (SRP)) from both streams.

Black poplar leaves were collected freshly abscised in autumn 2016 from the riparian area of A stream and dried at room temperature until needed. The dried leaf sets were inserted into mesh bags (0.5 mm mesh size and with approximately 10–12 leaves) and transferred to the streams (23 mesh bags in each stream) along a 100 m reach.

As previous studies demonstrated that the conditioning process follows a unimodal pattern in which the palatability of leaves increases to a maximum within 2 to 6 weeks and then declines, we removed the leaf mesh bags from each stream after 20 days of conditioning [27,28]. Afterwards, the mesh bags were transferred to the laboratory and 945 leaf discs of 16 mm diameter were obtained with a cork borer, avoiding veins. We repeated this process twice (with a week in between) to supply similar food conditions to the shredder (see below).

### Leaf litter quality after 14 days of conditioning

We used a set of leaf discs from each stream to characterize the initial communities of aquatic hyphomycetes. To induce conidial release from the hyphomycetes, five leaf discs with six replicates per stream were placed into 10 Erlenmeyer flasks with 60 ml of dechlorinated water at 14°C under 12 h light: 12 h dark conditions for 48 h. We aerated the Erlenmeyer flasks from the bottom by a continuous airflow to create turbulence that kept the leaf discs in continuous motion [29]. To determine the initial communities of aquatic hyphomycetes in the leaf discs, we filtered the suspensions of spores through 5-μm-pore size membrane filters (Cellulose Nitrate Membrane Filters, Whatman) and stained the filters with one drop of Trypan Blue solution containing lactic acid. For all the samples, we filtered the same volume (5 ml) of the suspensions of spores. Then, we used fields for identify and count the spores. First, we scanned the surface of the filter under the light microscope (400x), then we counted and identified all conidia and if they were very numerous, we counted the conidia in 20–30 randomly chosen microscope fields. We expressed the result as number of conidia per mL. Furthermore, based on these results we created a presence or absence conidia matrix [30].

The ergosterol concentration in the leaf discs was determined as a proxy for fungal biomass [31]. Five frozen leaf discs per five replicates per stream were lyophilized and weighed to determine the dry mass. We performed the lipid extraction and saponification using 0.14 M KOH methanol (8 g L^−1^) at 80°C for 30 min in a shaking water bath. The extracted lipids were purified using solid-phase extraction cartridges (Waters Sep-Pak, Vac RC, 500 mg, tC18 cartridges, Waters Corp., Milford, MA, USA), and ergosterol was eluted using isopropanol. We used high pressure liquid chromatography (HPLC) to detect and quantify the ergosterol by measuring the absorbance at 282 nm. We used a Jasco HPLC system (USA) equipped with a Gemini-NX 5 μm C18 250 × 4.6 mm column (Phenomenex, UK). The mobile phase was 100% methanol, and the flow rate was set to 1.2 ml min^−1^. Finally, we converted the ergosterol into fungal biomass using a conversion factor of 5.5 mg of ergosterol per gram of fungal mycelium [32]. We expressed the results in mg of fungal biomass per gram of dry mass leaf litter.

To determine the total lipid content in the leaf, we lyophilized five frozen leaf discs per five replicates per stream and homogenized them with an ultrasonic homogenizer (200 W, 24 kHz; Hielscher Ultrasonics GmbH, Teltow, Germany). We performed the lipid extraction with a monophasic solution of chloroform and methanol (2:1, v/v). Then, using a biphasic solution (chloroform and distilled water), we separated the phases, and after one night at 50°C, we analysed the total lipid content using the colorimetric sulpho-phospho-vanillin method [33]. We expressed the results as the percentage of total lipid.

Finally, another set of five leaf discs per five replicates from each stream was dried and ground into a fine powder to analyse the nitrogen (N) and carbon (C) concentrations using a Thermo Element Analyzer 1108 (Thermo Scientific, Milan, Italy). We expressed the results in terms of C:N molar ratios.

### Shredder

We collected individuals of *Potamophylax latipennis* (Trichoptera: F. Limnephilidae, Curtis, 1834) in stream A in February 2017 and transported them to the laboratory in plastic containers containing stream water and sand. To acclimatize the shredders to the laboratory conditions, they were maintained in declorinated water with food (leaves from the river) provided ad libitum for three days before starting the experiment. The temperature was maintained at 14°C. On the third day, the shredders were starved, which allowed evacuation of their gut contents. Once the shredders were acclimatized, we sorted 86 shredders with similar size. To calculate the average initial larval weight, average initial head width and the average initial lipid content, we separated thirty-six individuals we measured and frozen them at -80°C, lyophilized, and weighed (initial head width = 1.79 ± 0.03 mm; initial larvae dry mass = 0.031 ± 0.07 mg, n = 36).

### Microcosm setup

**Fig 1:**
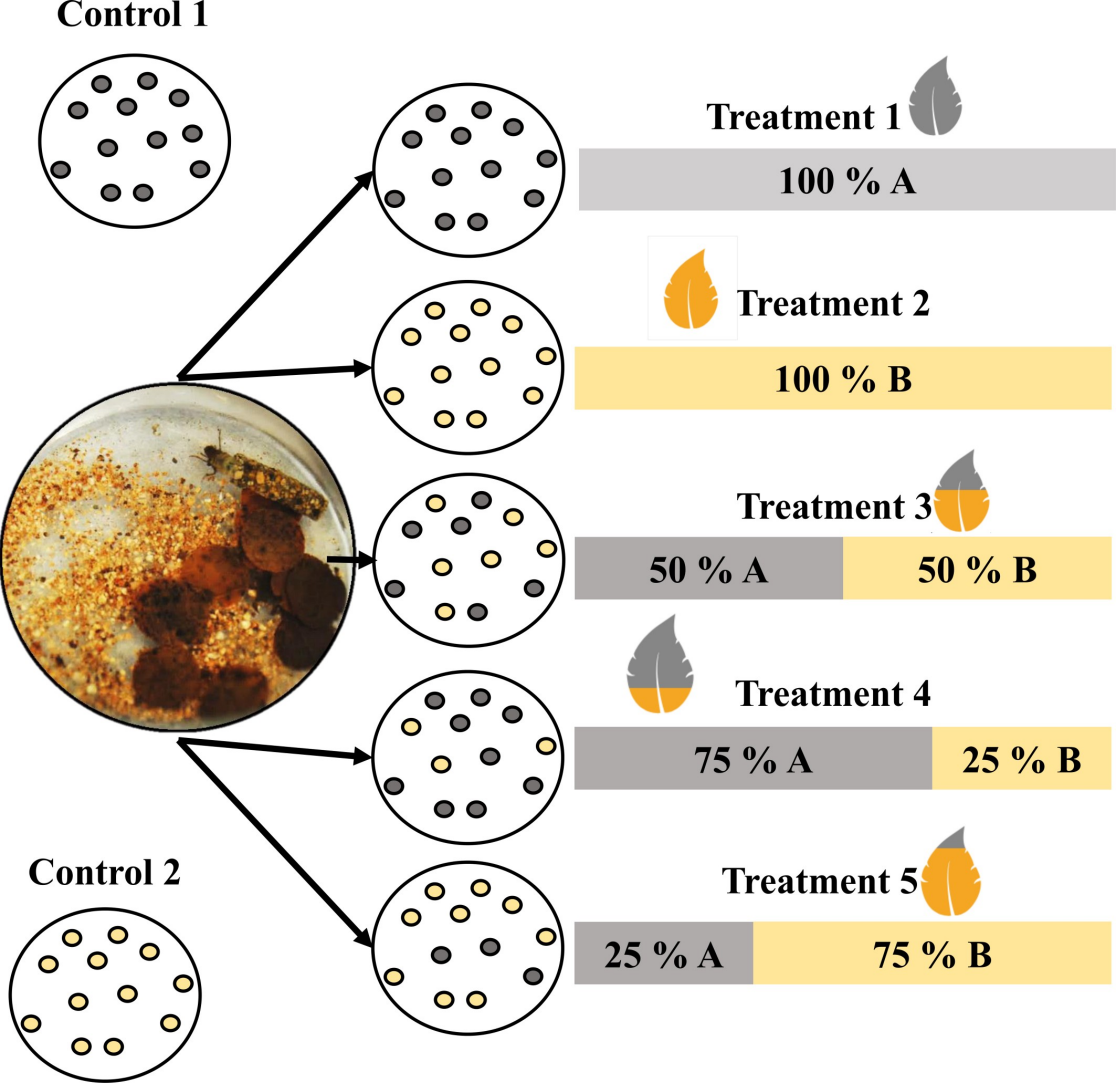
Experimental design. A and B refer to the leaf discs from the permanent and intermittent streams, respectively. Each microcosm contained individual shredders and 12 leaf discs. Ten microcosms were used per treatment.

We allocated the shredders individually into fifty glass microcosms (8.5 cm diameter and 8.3 cm height) with 60 ml of dechlorinated water. We added 12 leaf discs in each microcosm according to five different treatments: Treatment 1 (t1), ten microcosms with leaf discs from only stream A; treatment 2 (t2), ten microcosms with leaf discs from stream B; treatment 3 (t3), ten microcosms with equal proportions of leaf discs from streams A and B; treatment 4 (t4), ten microcosms with 75% of the leaf discs from stream A and 25% of the leaf discs from stream B; treatment 5 (t5), ten microcosms with 25% of the leaf discs from stream A and 75% of the leaf discs from stream B. In mixture treatments (from t3 to t5), discs from each type of leaves (A or B) were marked with colour pins. In addition, twenty microcosms containing leaf discs (ten with leaf discs from only stream A and ten with leaf discs from stream B) were maintained without shredders to serve as the controls for the loss of leaf not attributable to consumption (Fig 1).

We provided each microcosm with ash sand from the same riverbed (previously burnt at 450°C for 4 h) to allow larvae to build their cases. We aerated the microcosms by a continuous airflow under 12 h light: 12 h dark photoperiod conditions at 14°C in incubator rooms. The experiment lasted 2 weeks. Every three days during the experiment, we controlled the NH_4_ concentration of each microcosm (Tetra Test NH_3_/NH_4_, Tetra GmbH, Germany) and the water level. If the water level was below 60 ml, we added dechlorinated water until that level was reached. At the end of the first week, we replaced the water and leaf discs from those colonized one week later in the streams. All leaf discs were kept frozen at -80°C until analysis.

### Shredder consumption and growth

We determined the shredder consumption (C, mg) at the end of the experiment in each treatment as follows:

$$C=\sum_{k=1}^{2}\left(Li-Lf\right)$$ (1)

where *k* is the number of weeks of the experiment, and *Li* and *Lf* are the initial and final dry masses (hereafter DM, mg) of leaf discs for each week of the experiment, respectively, corrected by the DM leaf loss in the control microcosms without shredders of the respective treatments.

We calculated the relative consumption rate per larvae (RCR, mg leaf DM mg ^-1^ larval DM day ^-1^) as follows:

$$RCR=\frac{C/T}{w}$$ (2)

where *T* is the time for the entire feeding period (14 days) and *w* is the average of the larval DM (mg) at the beginning and end of the experiment.

We calculated the instantaneous growth rate (IGR, mm d^-1^) and the relative growth rate (RGR, mm mm ^-1^ d^-1^) using the head width (HW) of the larvae [12] as follows:

$$IGR=\frac{\ln(HWf)-\ln\left(HWi\right)}{T}=\frac{ln(\frac{HWf}{HWi})}{T}$$ (3)

and

$$RGR=\frac{HWf-HWi}{\left(HWf*T\right)}$$ (4)

where *HWf* and *HWi* are the final and initial head width (mm), respectively and *T* is the time for the entire feeding period (14 days).

We measured the metabolism via the oxygen consumption for nearly 10 min and corrected the measurement by the individual dry weight (mg O_2_ L^-1^ g DM^-1^ min^-1^). Measurements were made with an optical oxygen microsensor adapted to a 20 ml glass vial (Fibox 4 PreSens, Regensburg, Germany) filled with the oxygen-saturated water in which the shredder had been introduced. The oxygen concentration was recorded every 5 seconds for 10 min [34].

Finally, as a proxy for their body condition, we analysed the total lipid content of each shredder, expressed as a percentage of invertebrate DM. At the end of the experiment, each individual was frozen separately at -80°C. The protocol was similar to that for the leaves. We quantified the lipid content by spectrophotometry after digestion with H_2_SO_4_ (100°C) and comparison against a cholesterol standard [9].

### Data analysis

To characterize the stream hydrology in the intermittent stream, we used the daily variation of the streambed temperature corrected for the barometric pressure and air temperature. This daily variation was determined as the difference between the maximum and minimum temperature per day and the daily higher rate of change per hour. We performed a fifth-order moving average to smooth daily differences. To test the differences in water temperature, water concentrations of nutrients (nitrate, nitrite, ammonium and soluble reactive phosphorous (SRP)) from both streams, we performed a two samples t-test at the 95% confidence interval level.

To analyse the effects of flow regime on the leaf litter quality (chemical composition of leaf litter) and richness of aquatic hyphomycetes between streams A and B, we performed two sample t-tests at the 95% confidence interval level. To test differences between the composition of the conidia produced by the aquatic hyphomycetes colonizing the leaf litter from streams A and B, we used a multivariate generalized linear model (MANYGLM model, mvabund R package) due to it is a flexible and powerful framework for analysing abundance data and show a better power properties than distance-based methods [35]. Indicator taxa were defined for each stream class (A and B) using the indicator species analysis (IndVal) of Dufrene and Legendre [36]. This analysis generates an indicator value index (IV) for each species (based on the presence or absence of a spore matrix) and stream class. The indicator calculation is based on the specificity (maximum when the species occurs in only one stream) and fidelity (maximum when the species is present in both streams). To perform the tests, we used the packages vegan, mvabund, labdsv and ade4 in R.

To evaluate the differences between treatments in the endpoints measured in the larvae, we first checked the outliers of the data, the variable distributions (skewness) and the assumption of normality (Bartlett and Shapiro test). For variables that did not fulfil the assumptions of normality, we transformed the original data using a square root transformation for RCR and total lipid and a log_10_ (x+1) transformation for RGR and IGR. We performed one-way ANOVA using the treatment as the fixed effects factor with the car and sandwich packages in R. We validated the model visually by assessing the distribution of residuals for normality and homoscedasticity [37]. When the null hypothesis was rejected, we performed post hoc Tukey pairwise comparisons using the multcomp package in R.

Finally, to analyse the effect of leaf quantity on feeding preferences of *P*. *latipennis*, we performed a one sample t-test for the three-mixture treatment to compare the observed consumption of A leaf discs to the total consumption and the expected value, considering the last one as the real proportion of A leaf discs at each treatment (50%, 75% and 25%).

All the statistical analyses were performed using the R statistical software version 3.4.1 [38], with the significance level set at p < 0.05 for all tests. The datasets used in this study are available in S1 Table.

## Results

The intermittent stream presented a summer drought with 46 dry days and an average temperature of 10.06°C (± 1.99), whereas the permanent stream presented an average temperature of 10.70 (± 1.90) (differences were not significant, t-test, t_6_ = -0.34, p-value = 0.741). The intermittent stream SRP was 0.008 ppm (± 0.001), and the permanent stream SRP was 0.013 ppm (± 0.002) (t-test, t_6_ = -0.94, p-value = 0.367). The total dissolved inorganic nitrogen (DIN = nitrite + nitrate + ammonia) concentration was 0.24 (± 0.07) in the intermittent stream and 0.41 ppm (± 0.13) in the permanent stream (t-test, t_6_ = -1.92, p-value = 0.086).

### Effects of flow regime on the leaf litter quality

The flow regimen significantly affected the quality of the leaf litter after 20 days of conditioning in streams A and B. The quality differed on lipid content, fungal biomass and aquatic hyphomycetes richness (Table 1) being higher for the leaf litter conditioning in stream A.

**Table 1:** Means ± SEM of the initial chemical leaf litter composition (n = 5) and aquatic hyphomycetes richness (n = 6) and t-tests results for the quality of the leaves in both treatments (A and B).

	Mean (± SEM)		Statistics	
Variable	Permanent (A)	Intermittent (B)	t_8_	p-value
Total lipid (%)	4.13 (± 0.07)	3.36 (± 0.02)	9.45	**<0.001**
C:N	63.5 (± 2.9)	51.2 (± 1.5)	2.42	0.051
Fungal Biomass (mg FB/g DM)	33.71 (± 4.02)	17.68 (± 5.09)	4.30	**0.004**
Aquatic hyphomycetes richness	12 (± 0.4)	5 (± 0.6)	8.87	**<0.001**

The structure of the species in the initial aquatic hyphomycetes communities associated with the leaf litter in streams A and B were significantly different (MANYGLM, p = 0.001). IndVal analysis revealed that the species *Heliscus submersus*, *Alatospora acuminta*, *Tetrachaetun elegans* and *Lemonniera aquatica* were significantly different, and the species *Fusarium* sp. and *Articulospora tetracladia* were marginally significantly different between the streams. Moreover, *Cilindrocarpon* sp., *Alatospora acuminata*, *Tetracladium setigerum*, *Tetracladium marchalianum*, *Anguillospora longuissima*, *Tricladium chaetocladium*, *Lemonniera aquatica* and *Clavariopsis aquatica* appeared in only stream A, whereas *Dendrospora* sp. appeared in only stream B (Table 2). However, MANYGLM analysis also revealed that the dominant species in both streams *Flagellospora curvula*.

**Table 2:** Results of the indicator species analysis (IndVal), maximum IV significance (IV is the individual value), associated stream class for each species and the frequency of appearance (A and B, permanent and intermittent streams).

Species	Class	IV	p-value	A	B
*Flagellospora curvula*	1	0.500	1	100	100
*Heliscus lugdunensis*	1	0.600	0.461	100	66.7
*Heliscus submersus*	1	0.750	**0.012**	100	16.6
*Tetrachaetum elegans*	1	0.857	**0.024**	100	16.67
*Fusarium* sp.	2	0.694	0.062	16.6	83.3
*Cylindrocarpon* sp.	1	0.667	0.075	66.6	0
*Alatospora acuminata*	1	1.000	**0.007**	100	0
*Articulospora tetracladia*	1	0.694	0.074	83.3	16.6
*Lemonniera pseudofloscula*	1	0.521	0.567	83.3	50
*Lemonniera aquatica*	1	0.667	**0.049**	66.6	0
*Dendrospora* sp.	2	0.167	1	0	16.6
*Tetracladium setigerum*	1	0.167	1	16.6	0
*Tetracladium marchalianum*	1	0.500	0.205	50	0
*Anguillospora longissima*	1	0.333	0.453	33.3	0
*Tricladium chaetocladium*	1	0.167	1	16.6	0
*Clavariopsis aquatica*	1	0.333	0.446	33.3	0

### Effects of leaf litter quality and quantity on *P*. *latipennis*

Differences in the leaf litter quality between streams A and B significantly affected the consumption and growth of *P*. *latipennis* (Table 3).

**Table 3:** Results of one-way ANOVA (factor treatment) of the effects of leaf quality on consumer consumption and growth.

Variable	Treatment		
	df	F	P
Total Consumption (mg leaf DM)	4	4.481	**0.004**
RCR (mg leaf DM mg larval DM ^-1^ day ^-1^)	4	4.317	**0.005**
IGR (mm d^-1^)	4	5.622	**0.001**
RGR (mm mm^-1^ d^-1^)	4	5.074	**0.002**
Total Lipids (%)	4	2.877	**0.035**
Oxygen Consumption (mg L^-1^ mgDM^-1^ s^-1^)	4	3.926	**0.041**

N = 50 (10 replicates per treatment) except for oxygen consumption, where n = 15 (three replicates per treatment).

**Fig 2:**
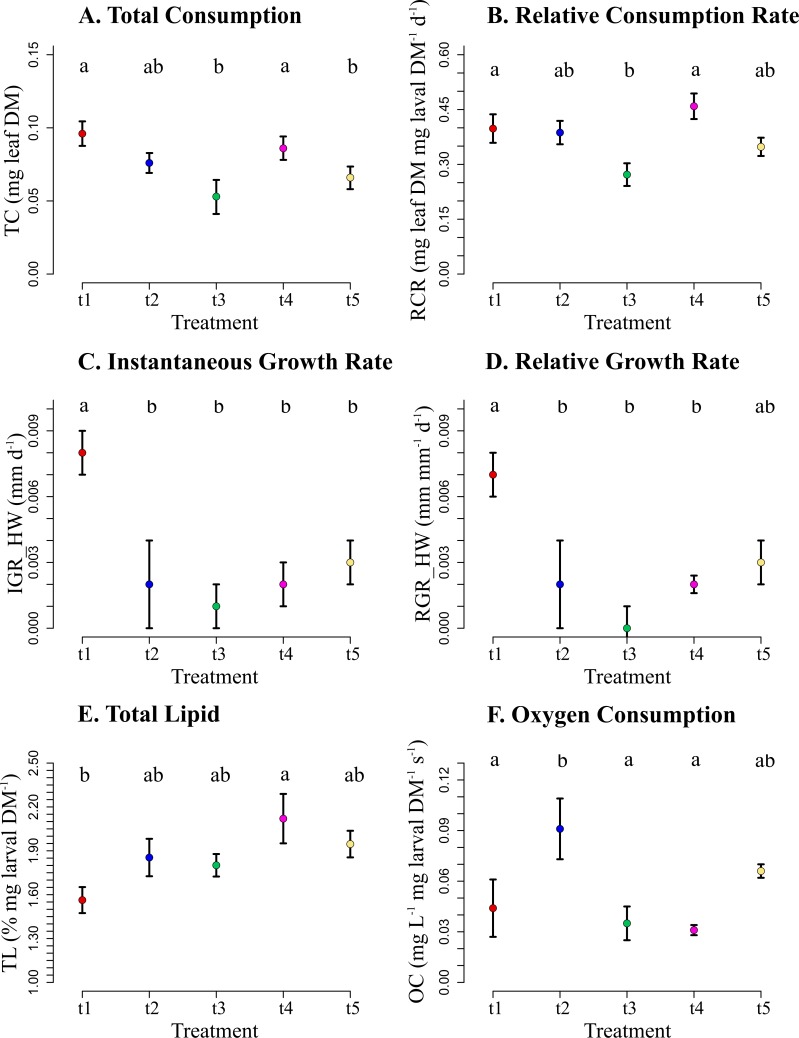
Shredder consumption and growth. Total consumption (A), relative consumption rate (RCR) (B); instantaneous growth rate of the head width (IGR) (C); relative growth rate of the head width (RGR) (D); total lipid content (E) and, oxygen consumption (F) of *P*. *latipennis*, where t1 = 100% A, t2 = 100% B, t3 = 50% A and 50% B, t4 = 75% A and 25% B and t5 = 25% A and 75% B. The different letters indicate significant differences (Tukey HSD post hoc test, p< 0.05) among treatments for each variable.

Total consumption was significantly higher in treatments with a higher proportion of A leaf discs, i.e., t1 and t4 treatments with 100% and 75% of A leaf discs, respectively. Post hoc comparisons also showed significant differences (Tukey HSD, p<0.05) between treatments t1 and t4 and treatments with a lower proportion of A leaf discs, i.e., t3 and t5 treatments, with 50% and 25% of A leaf discs, respectively (Fig 2A). The RCR was also significantly lower in treatment t3 (50% of A leaf discs) than in the t1 and t4 treatments (Tukey HSD, p<0.05; Fig 2B).

The IGR based on head larvae width was 0.019 mm ± 0.001 per day for t1 (Fig 2C), whereas for the rest of the treatments, the head width showed lower IGRs (mean ± SEM, t2 = 0.005 ± 0.004, t3 = -0.000 ± 0.003, t4 = 0.005 ± 0.001 and t5 = 0.007 ± 0.001), and significant differences were found between treatments t1 and the others (Tukey HSD, p<0.05). The RGR (Fig 2D) were significantly different between t1 and t2 (Tukey HSD p<0.05). Therefore, when the larvae were fed with leaf discs of higher quality (t1, 100% A leaf discs), their relative growth was higher (mean ± SEM, t1 = 0.017 ± 0.001, t2 = 0.004 ± 0.001), and even in the RCR, no significant differences were observed (Fig 2B). Moreover, there were also significant differences between t1 and t3, and surprisingly, between t1 and t4 (Tukey HSD p<0.001 and p = 0.010, respectively). The t4 treatment showed higher consumption but lower growth. Larvae of this treatment showed a greater proportion of lipids than t1 (Tukey HSD, p = 0.019) (Fig 2E).

The metabolism of the shredder larvae was significantly higher in t2 (100% B leaf discs) than in the other treatments (Tukey HSD, p<0.005) except for t5, in which 75% of the leaves were from B (mean ± SEM, t1 = 0.04 ± 0.01; t2 = 0.09 ± 0.01; t3 = 0.03 ± 0.01; t4 = 0.03 ± 0.02 and t5 = 0.06 ± 0.01) (Fig 2F). No mortality was observed during the experiment.

When leaf discs from stream A and B were offered simultaneously in different quantities to the shredders (mixture treatments: t3, t4 and t5), the shredders showed a tendency to select leaf discs from A (t-test, t_14_ = 2.184, p = 0.047).

**Fig 3:**
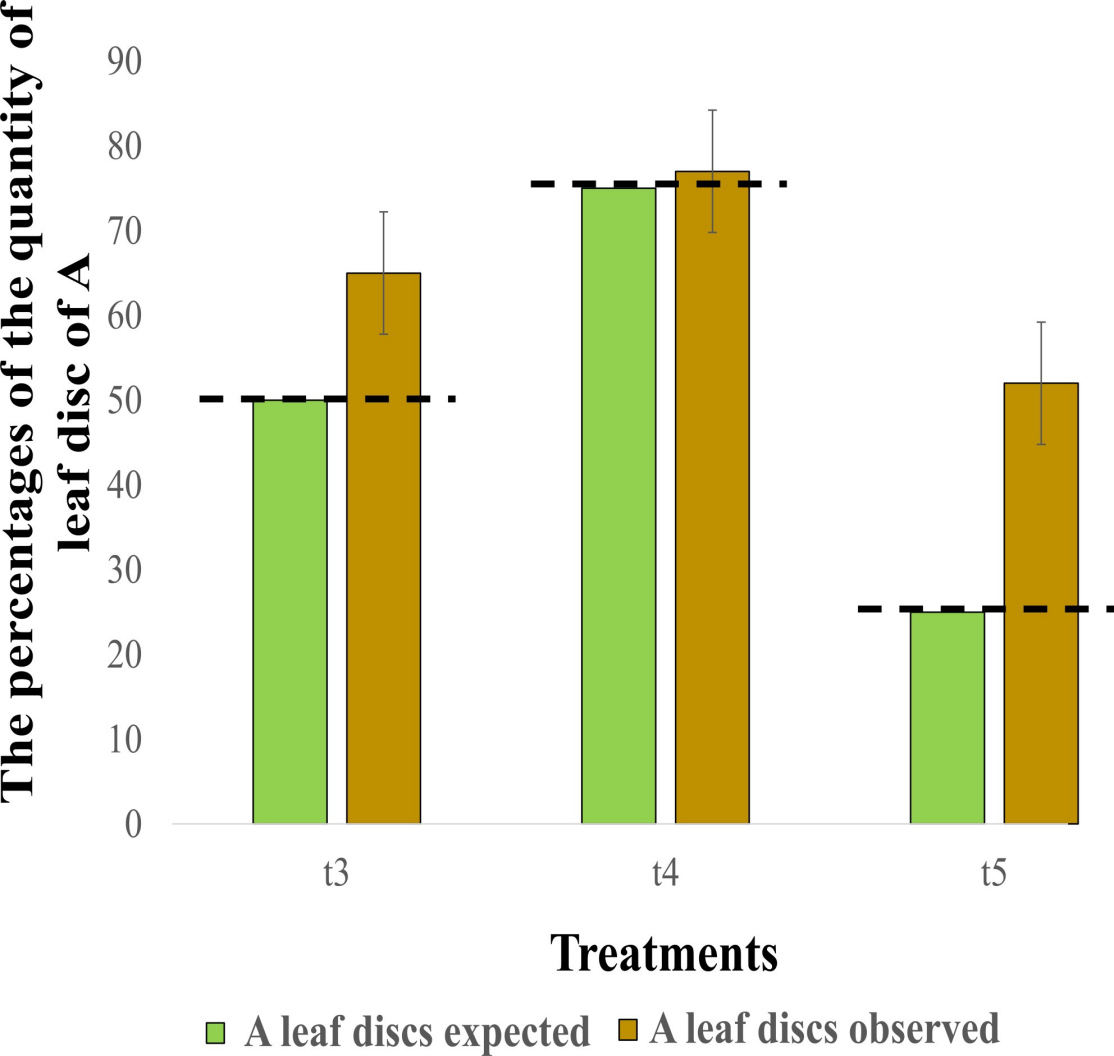
The observed consumption (black bars) of A leaf discs compared with the expected consumption (grey bars). The expected A leaf discs as the initial proportion of A discs at each treatment: t3 = 50%, t4 = 75% and t5 = 25%). N = 5 (t3, t4) and N = 4 (t5).

The percentages of the quantity of A leaf discs consumed regarding the consumption expected at each treatment according to the quantity of each type of leaf disc were mostly greater than the expected consumption at each treatment (Fig 3). In t3, the larvae consumed 65% of A leaf discs, whereas the expected amount was 50% (15% more than expected), while in t4, larvae consumed 77% and 75% was expected (2% more than expected), and finally, the t5 larvae consumed almost all the A leaf discs (27% more than expected). When A leaf discs were offered below 50% (treatment 5), the shredder fed selectively A leaf discs (t-test, t_5_ = 2.5379, p = 0.042).

## Discussion

Our results showed that flow intermittency reduces the quality of leaf litter in terms of fungal richness and biomass and lipid content (*Objective 1)*. In addition, these changes in food quality influenced the consumption rates (i.e., the leaf litter most consumed were those conditioned under permanent flow) and growth of the shredder (*Objective 2*). Finally, *P*. *latipennis* fed selectively on higher quality leaves; although its availability (quantity) was lower (*Objective 3*).

### Effects of flow regime on leaf litter quality

This study pointed out that flow regime influenced the leaf litter quality by means of changes in fungal colonization. Permanent flow allowed the continuous colonization of leaf litter, resulting in higher fungal richness, biomass and lipid content than leaf litter conditioned under intermittent flow conditions.

According to Suberkropp et al. [39], hyphomycete richness affects the palatability of the leaf resources, as a higher leaf litter quality is associated with fungal composition and richness. Several studies reported that shredders preferentially fed on well-conditioned leaf litter [12,15], probably related to the characteristics of the fungi themselves (high nutritional value, [40]) and to the chemical modifications of leaf litter by fungi [10]. Different fungal species have different degradative capabilities that make leaf litter more palatable. Previous studies [39,40] demonstrated that *A*. *acuminata*, *C*. *aquatica*, *F*. *curvula*, *L*. *aquatica and T*. *marchalianum* had the capacity to produce enzymes that degraded polygalacturonic acid, xylan and carboxymethyl cellulose. Our results showed that the abundances of most of these species (except *F*. *curvula*) were higher on leaf litter conditioned under permanent than intermittent flow condition. Another species, *T*. *elegans*, has similar enzyme capabilities [41], and this species was more abundant in the permanent stream. Furthermore, *H*. *lugdunensis* has been reported as a fungal colonizer preferred by shredders [42], and this specie appears in a higher abundance in leaf litter colonized under continuous flow. All of these data reinforce the idea that permanent flow conditions promoted a higher fungal richness on leaf litter, and therefore, a higher litter quality than under intermittent flow conditions. To further explore this idea, we suggest that additional studies should be conducted using molecular analysis to evaluate the roles of other low-abundance fungal species that might be relevant in terms of leaf palatability. Such studies will provide knowledge on specific fungal traits and enzymatic activities.

In addition to fungal richness, our results also pointed out that higher leaf-associated fungal biomass occurred under permanent than intermittent flow conditions, which is consistent with previous studies [43–45]. While flow disruption constrained and retarded fungal growth and colonization, permanent flow stimulated the sporulation process and supplied a continuous source of fungal spores to leaf litter [10]. A higher fungal biomass is related to a higher litter quality and palatability, attributable to an enrichment of N due to the uptake and immobilization of this element from the water column by fungal communities [46].

The lower fungal biomass found under intermittent flow conditions also influenced the lipid content, as demonstrated in other studies (e.g., [9,19,45]). Flow intermittency determines a reduction in the total and essential fatty acids in leaf litter [19,47], which influences its quality. Müller-Navarra et al. [48] found that the contents of lipids, such as fatty acids, including polyunsaturated fatty acids, is essential and can limit consumer growth, reproduction, neural development and trophic transfer efficiency. In accordance, the higher total lipid concentration found in leaf litter colonized in the permanent stream led to a better quality [49,50] for consumers.

Finally, molar C:N ratios are considered an important indicator of the nutritional value of food resources due to the positive correlation between nitrogen content and shredder preferences [51]. Unfortunately, in our results, we did not find significant differences in the C:N ratios for leaf litters conditioned in the two streams.

### Effects of the leaf quality and quantity on consumer consumption and growth

Several studies have shown the importance of nutritional quality of the leaf litter resource for consumer feeding preferences and growth. Gonçalves et al. [5,15] highlighted the importance of fungal composition and richness on shredder feeding rates, and Arsuffi & Suberkropp [52] showed the importance of lipids and proteins for stimulating shredder consumption, as shredders cannot synthesize these components and must therefore acquire them from their diet [13,53].

The results of our experiment showed that despite the differences in leaf quality, the total consumption and RCR between the leaf litters conditioned in both streams were not significantly different. The similar consumption rates observed in our study between the t1 and t2 treatments (100% leaf discs from A and B, respectively) could be related to the fungal composition of the leaf litter in both streams, among other factors. As indicated previously, two fungal species reported as being highly palatable to shredders (*F*. *curvula and H*. *lugdunensis)* were abundant in the leaf litters conditioned in both streams [42,43,52,54]. However, when we simultaneously offered leaf discs from both streams (treatments t3, t4 and t5), shredders consumed less when A leaf discs were in a lower proportion (t3, t5; 50% and 25%, respectively) in relation with t1 and t 4 (100% and 75%, respectively). There was a preferential selection of A leaf discs in all mixture treatments, as demonstrated by the feeding preference results (Fig 3). The higher fungal biomass and lipid content of the A leaf discs together with their fungal composition stimulate the shredder selection of these leaves in mixture treatments.

Fungal biomass accrual on leaves tends to increase the leaf N content, and the enzymatic maceration of leaves by the fungal community results in smaller and less refractory plant polymers, both processes making leaf resources more palatable to shredders [55,56]. Nevertheless, other studies show that a high fungal biomass does not necessarily imply a higher palatability of leaves, suggesting that shredder feeding depends on other characteristics, such as the leaf toughness, nutrient content, presence of mycotoxins and adaptation of shredders to those chemicals [43,57].

The similar consumption rates between A and B leaves did not translate to similar growth rates. The RGR and IGR were lower when only B leaf discs were offered. This result suggests that the consumption rate of B leaf discs was not sufficient to achieve similar growth. Consumers have two ways to compensate for the limitations of a poor resource quality, increasing consumption (feeding compensation [58]) or increasing assimilation rates, for example, by enhancing the retention time in their guts [59]. In general, the treatments with leaf mixtures also showed significantly lower growth rates regarding t1 with the exception of t5 for RGR. Our shredder mainly selected A leaf discs in mixture treatments, but the lower availability and/or the presence of less palatable leaf discs from stream B also limited its growth rate.

Food quality affects energy allocation (lipid storage). According to Flores et al. [58], the larvae fed poor-quality resources allocated a higher proportion of lipids to their body conditions than to growth. Larvae fed leaves of the poorest quality (from stream B) tended to allocate more lipids than larvae fed leaves of the richest quality (from stream A). Nevertheless, we did not find significant differences.

Finally, our results showed that the leaf quality affected the basal metabolism of the larvae. The basal metabolic rate determines the energetic cost of living, and after meeting the baseline energy requirements, shredders tend to allocate excess energy to other functions, such as growth and reproduction. The larvae fed leaf discs from stream B showed the highest oxygen consumption rate. This higher metabolism leads to lower energy being invested in growth, as shown in our results [59].

Consumers tend to maximize their feeding preferentially on food resources that are energetically most profitable [20]. They meet their elemental composition requirements to optimize their growth and reproduction, feeding preferentially on high-quality resources [60,61], and our findings are consistent with these statements. While the response of shredders has been strongly related to resource quality, what happens when high-quality resources are scarce remains in question. Other questions remaining include whether consumers actively search for high-quality resources even though they are the least abundant or whether they prefer to consume without selection and exert a more efficient assimilation to maintain homeostasis. Cruz-Rivera & Hay [62] suggested that resource selection seems to be related to the mobility of organisms. Our results suggest that when mobility is not a handicap, shredders seemed to actively select the food of better quality based on the quality properties despite its lower abundance, although this can limit their growth. We hope this finding stimulates future research to explore how mobility and resource availability interact in shredders.

In the context of increasing global water demand and aridification, flow intermittency will become more frequent, leading to drastic changes in food quality and quantity in rivers. Our findings demonstrate that such changes affect the fungal colonization of leaf litter, reducing several litter quality properties and ultimately affecting shredder consumption rates, growth and feeding selections. These responses could therefore potentially threaten the entire fluvial food web. These results provide a better understanding of the effects of changes in flow conditions on ecosystem functioning (leaf litter processing) in rivers and warn of the importance of guaranteeing the natural hydrological dynamics via a better management of water use.

## Supporting information

S1 Table Consumer consumption and growth data. (PDF)
