# Supplementary material for: Quality and quantity of leaf litter: Both are important for feeding preferences and growth of an aquatic shredder
Source: PLoS One. 2018 Dec 12;13(12):e0208272. doi: 10.1371/journal.pone.0208272 (PMC6291097; doi:10.1371/journal.pone.0208272)
Supplement: S1 Table — (PDF) [file pone.0208272.s001.pdf]

Supporting information

S1 Table. Consumer consumption and growth data.

	Mean (±SEM)				
Variable	**t1**	**t2**	**t3**	**t4**	**t5**
Total Consumption (mg leaf DM)	0.096 (±0.008)	0.076 (±0.006)	0.053 (± 0.010)	0.086 (±0.006)	0.066 (±0.007)
RCR (mg leaf DM mg larval DM $^{-1}$ day $^{-1}$)	0.171 (±0.034)	0.157 (±0.026)	0.081 (±0.019)	0.221 (±0.033)	0.125 (±0.019)
IGR (mm d^{-1})	0.019 (±0.001)	0.006 (±0.004)	0.001 (±0.003)	0.005 (±0.001)	0.007 (±0.001)
RGR mm mm^{-1} $d^{-1)}$	0.017 (±0.001)	0.005 (±0.004)	0.001 (±0.003)	0.005 (±0.001)	0.006 (0.001)
Total lipids (%)	2.489 (±0.271)	3.536 (±0.494)	3.291 (±0.267)	4.725 (±0.745)	3.862 (±0.357)
Oxygen Consumption (mg L^{-1} mg DM^{-1} $s^{-1)}$	0.044 (±0.016)	0.091 (±0.018)	0.035 (±0.010)	0.031 (±0.002)	0.066 (±0.004)
